# Association between carotenoid intake and periodontitis in diabetic patients

**DOI:** 10.1017/jns.2023.116

**Published:** 2024-02-28

**Authors:** Fengli Li, Ge Wang, Yujie Zhang

**Affiliations:** 1 Department of Maxillofacial Surgery, Shungeng Branch, Jinan Stomatological Hospital, Jinan, Shandong, People’s Republic of China; 2 Department of Conservative and Endodontic Dentistry, East Branch, Jinan Stomatological Hospital, Jinan, Shandong, People’s Republic of China; 3 Department of Orthodontics, Shanda North Road Branch, Jinan Stomatological Hospital, Jinan, Shandong, People’s Republic of China

**Keywords:** α-carotene, diabetes, dietary carotenoids, lutein and zeaxanthin, NHANES, periodontitis

## Abstract

This study aimed to evaluate the association between dietary carotenoid intake and periodontitis in diabetic patients. Data on diabetic patients were collected from the National Health and Nutrition Examination Survey (NHANES) 2009–2014 for this cross-sectional study. Dietary intake of carotenoids was assessed through the first 24-hour dietary recall interview. Full-mouth periodontal examinations were conducted by trained dental examiners. Subgroup analysis was conducted in terms of age, gender, the number of missing teeth, cardiovascular disease, smoking, and anti-diabetic drugs. Totally 1914 diabetic patients were included, with 1281 (66.93%) in the periodontitis group. After adjusting for age, gender, race, education, smoking, dental implants, hepatitis, and the number of missing teeth, α-carotene intake ≥55.82 mcg was associated with lower odds of periodontitis than α-carotene intake <55.82 mcg [OR = 0.70, 95% CI: 0.53–0.91, *P* = 0.010]; lutein and zeaxanthin intake ≥795.95 mcg was associated with decreased odds of periodontitis than lutein and zeaxanthin intake <795.95 mcg (OR = 0.75, 95%CI: 0.57–0.98, *P* = 0.039). The association between carotenoid intake and periodontitis varied across different subpopulations. In diabetes, dietary intake of α-carotene and lutein and zeaxanthin was inversely associated with the odds of periodontitis, which may facilitate clinical periodontitis management.

## Introduction

Periodontitis is a common chronic inflammatory disease, affecting over 40% of American adults^(1)^. Individuals with periodontitis have an increased risk of tooth loss and chewing dysfunction, which exerts a negative influence on their quality of life^(2)^. Diabetes is an independent risk factor for periodontitis, and the susceptibility of diabetic patients to periodontitis is elevated by about three times, which may be related to the oxidative stress and increased inflammatory levels caused by hyperglycaemia, thus causing damage to periodontal tissue^(3,4)^. Hence, it is necessary to explore factors associated with the occurrence and development of periodontitis in the diabetic population, and investigate potential prevention and control ways.

Dietary nutrition is a modifiable factor for periodontitis. Carotenoids are widely distributed fat-soluble pigments found in many fruits and vegetables, such as citruses, persimmons, carrots, and tomatoes^(5)^. Exiting studies found that lower intake of dietary carotenoids was related to higher severity of periodontitis, which may be attributed to the antioxidant properties of carotenoids^(6,7)^, but these studies have not made further analysis for diabetic patients. It is worth noting that the level of carotenoids in patients with diabetes is often low, suggesting that its increased consumption may be used to control the excessive oxidative stress induced by abnormal glucose metabolism^(8,9)^. Besides, animal experiment results showed that carotenoids supplementation (β-carotene, lycopene, and lutein) could improve oxidative stress and inflammatory status in patients with diabetes and its complications^(10–12)^. Nonetheless, there is still a lack of relevant research on the relationship between carotenoid intake and periodontitis in individuals with diabetes, which requires studies to improve the understanding of this relationship and promote management of periodontitis in diabetic patients.

The purpose of this study was to explore the association between dietary intake of carotenoids (α-carotene, β-carotene, β-cryptoxanthin, lutein and zeaxanthin, and lycopene) and periodontitis in the diabetic population by utilizing the data from the National Health and Nutrition Examination Survey (NHANES). This association was also evaluated in terms of age, gender, the number of missing teeth, cardiovascular disease, smoking, and anti-diabetic drugs.

## Materials and methods

### Study population

This study had a cross-sectional design, and used data on diabetic patients from 3 NHANES cycles (2009–2010, 2011–2012, and 2013–2014) which had the latest and consistent assessment of periodontitis. The NHANES is a series of multi-stage surveys conducted by the Centers for Disease Control and Prevention (CDC) to investigate the health and nutritional status of the nationally representative population in the United States (about 5,000 individuals annually), which combines interviews and physical examinations (https://www.cdc.gov/nchs/nhanes/about_nhanes.htm). Written informed consent to participate in this survey has been provided by participants. The NHANES is performed with permission from the NCHS Ethics Review Board (ERB) (https://www.cdc.gov/nchs/nhanes/irba98.htm). Since freely accessible and de-identified data from the NHANES were used, approval by the institutional review board was waived for this study. Individuals who (1) were diagnosed with diabetes; (2) were assessed by oral health exams for periodontal status; and (3) had measurement of dietary carotenoids. Edentulous patients were excluded from the current study. A maximum number of teeth was 28, and a minimum of teeth was 2.

### Diabetes

Individuals with Hb A1c (HbA1c) ≥6.5%, fasting glucose ≥126 mg/dL, serum glucose at 2 hours after a 75 g glucose load ≥200 mg/dL, self-reported diagnosis of diabetes, or self-reported use of insulin or other diabetes medication were regarded to have diabetes^(13–15)^.

### Dietary carotenoid intake

Dietary intake of α-carotene, β-carotene, β-cryptoxanthin, lutein and zeaxanthin, and lycopene was evaluated through the first 24-hour dietary recall interview, which was collected in-person in the Mobile Examination Center (MEC). The MEC dietary interview room provides a set of measurement guides (various glasses, bowls, mugs, etc.) for participants to report their food intake. These carotenoids were used as binary variables for analysis based on their median intake. Retinol intake was also measured as above. Intake of lutein and zeaxanthin supplements and lycopene supplements was also assessed via the first 24-hour dietary recall interview in the NHANES database, which was included in the intake of lutein and zeaxanthin, and lycopene, respectively.

### Periodontitis

Participants from the NHANES 2009–2014 aged ≥30 years underwent a full-mouth periodontal examination by trained dental examiners, since most of people aged ≥30 years were affected by periodontitis^(16)^. This full-mouth periodontal examination covered six sites per tooth in a maximum of 28 teeth. Periodontal probes were used to assess gingival recession and periodontal pocket depth at six sites per tooth, and clinical attachment loss was calculated^(17,18)^. This study divided periodontitis into no or mild periodontitis (no periodontitis group), and moderate or severe periodontitis (periodontitis group) according to the CDC-American Academy of Periodontology (AAP) definitions^(19)^: Mild: ≥2 interproximal sites with clinical attachment loss (CAL) ≥3 mm, and ≥2 interproximal sites with probing depth (PD) ≥4 mm (on different teeth) or 1 site with PD ≥5 mm; Moderate: ≥2 interproximal sites with CAL ≥4 mm (on different teeth), or ≥2 interproximal sites with PD ≥5 mm (on different teeth); Severe: ≥2 interproximal sites with CAL ≥6 mm (on different teeth) and ≥1 interproximal site with PD ≥5 mm. This classification was made to mitigate the risk of bias due to a potentially excessive prevalence of mild periodontitis in the population^(20)^, and individuals with mild periodontitis is not as significant as moderate or severe according to the CDC-APA classification.

### Covariates

The following covariates were obtained: age (years), gender, race, education, poverty-to-income ratio, smoking, drinking, physical activity, dental implants, diabetic retinopathy, chronic kidney disease, hypertension, dyslipidaemia, cardiovascular disease, hepatitis, autoimmune disease, BMI (kg/m^2^), waist circumference (cm), white blood cell count (1000 cells/μL), total energy (kcal), total fat (gm), the number of missing teeth, frequency of using dental floss, antibiotics, and anti-diabetic drugs. Race was classified into non-Hispanic White, non-Hispanic Black, and other races^(21)^. Education level was divided into less than high school, high school graduate/general educational development (GED) or equivalent, and above high school^(22)^. Drinking status included no drinking, drinking <1 time per week, and drinking ≥1 time per week^(23)^. Physical activity was converted into energy consumption, and classified into <450 MET·min/week, ≥450 MET·min/week and unknown^(24)^. Cardiovascular disease was defined as self-reported CHD, angina, heart failure, heart attack, stroke, or use of cardiovascular medication^(25)^. Autoimmune disease was defined as rheumatoid arthritis, inflammatory bowel disease, ankylosing spondylitis, or thyroiditis. Total energy and total fat were estimated via the first 24-hour dietary recall interview, which also considered their intake from supplements. The number of missing teeth was divided into ≤ 5 and > 5. Frequency of using dental floss was classified into <3 times/week and ≥3 times/week.

### Statistical analysis

Measurement data were described as mean (standard error) [Mean (SE)], and the independent sample *t*-test was applied for comparison between two groups (no periodontitis group, and periodontitis group); enumeration data were reported as the number of cases and constituent ratio [*n* (%)], inter-group comparison was conducted using the Chi-square test, and the rank sum test was utilized for ranked data. Due to the high proportion of missing data in variables physical activity and poverty-to-income ratio, these missing data were classified as ‘unknown’. Other variables with missing data were filled using the chain equation multiple imputation method of random forest. Sensitivity analysis was carried to evaluate whether there were statistical differences between the data before and after imputation (Supplementary Table S1).

Weights are established in the NHANES to account for the complex survey design (including oversampling), survey non-response, and post-stratification adjustment to match total population counts from the Census Bureau. When a sample in the NHANES is weighted, it is representative of the civilian noninstitutionalized resident population in the United States (https://wwwn.cdc.gov/nchs/nhanes/tutorials/Weighting.aspx). Variables for weighting included SDMVPSU, SDMVSTRA and WTDRD1. Weighted univariate logistic regression analysis was used to explore the potential factors related to the odds of periodontitis. Then weighted logistic regression analysis was performed to investigate the association between intake of carotenoids and the odds of periodontitis. Model I was a univariate model; Model II, a multivariate model, was adjusted for age, gender, and race; Model III, a multivariate model, was adjusted for factors which were significantly associated with periodontitis in the univariate logistic regression analysis, i.e. age, gender, race, education, smoking, dental implants, hepatitis, and the number of missing teeth. The association between dietary carotenoid intake and periodontitis (mild or moderate, severe) was also assessed with adjustment for age, gender, race, education, smoking, dental implants, hepatitis, and the number of missing teeth. As a common treatment choice, dental implants were defined as teeth replaced with surgical implants, which may affect the odds of periodontitis. Subsequently, subgroup analysis was conducted in terms of age, gender, the number of missing teeth (≤5, 6–10, 11–15, >15), cardiovascular disease, smoking, and anti-diabetic drugs to assess whether the association between carotenoid intake and periodontitis differed in subpopulations. ORs and 95% CIs were estimated.

Python 3.9 (Python Software Foundation, Delaware, USA) was adopted for data cleaning and missing value processing, and SAS 9.4 (SAS Institute Inc., Cary, NC, USA) for model statistical analysis. All statistical tests were two-sided, and *P* < 0.05 was considered to be significantly different.

## Results

### Participant characteristics

**Fig. 1.**
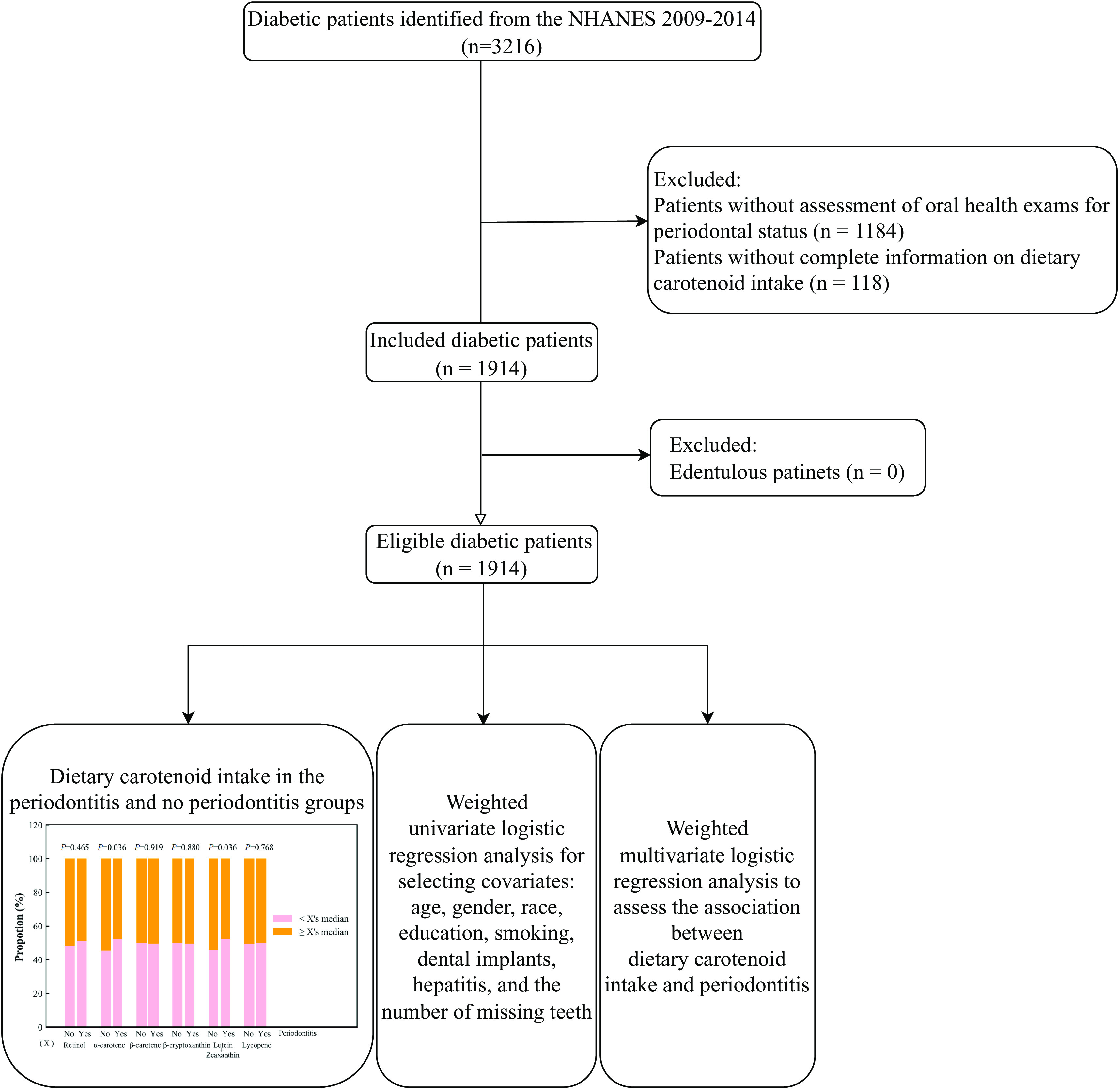
Flow chart of selecting eligible participants. NHANES: the National Health and Nutrition Examination Survey.

**Fig. 2.**
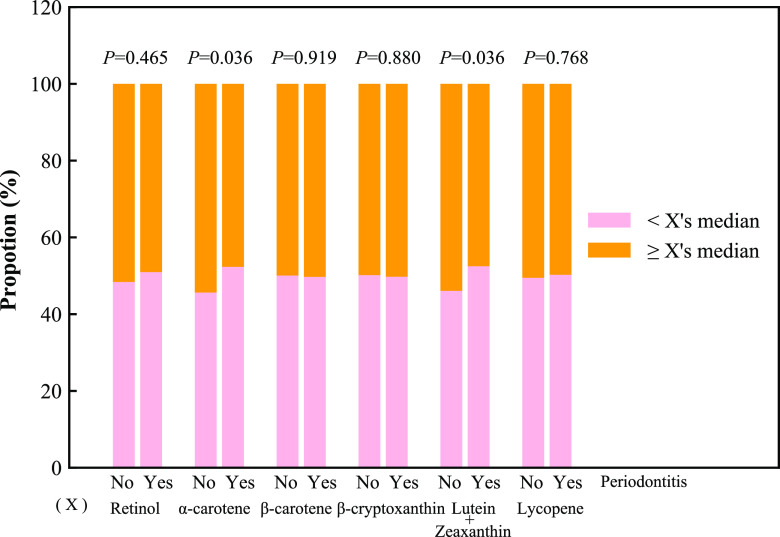
Dietary carotenoid intake of the periodontitis and no periodontitis groups.

A total of 3216 diabetic patients were identified. After excluding patients without assessment of oral health exams for periodontal status (*n* = 1184), and without complete information on dietary carotenoid intake (*n* = 118), 1914 patients were included in the end, with 633 (33.07%) in the no periodontitis group, and 1281 (66.93%) in the periodontitis group. Figure 1 shows the selection process of eligible participants. These patients had a mean age of 58.34 years, with 52.68% males and 47.32% females. Individuals in the no periodontitis group tended to be younger, female, non-Hispanic White, and non-smokers, have an education level above high school, a poverty-to-income ratio > 1.0, more physical activity, dental implants, no hypertension, no hepatitis, and ≤ 5 missing teeth, and use dental floss ≥3 times/week, in contrast to individuals in the periodontitis group (all *P* < 0.05). Dietary intake of α-carotene and lutein and zeaxanthin was higher in the no periodontitis group than that in the periodontitis group (both *P* < 0.05) (Fig. 2). Table 1 demonstrates the characteristics of the included diabetic patients.

**Table 1.** Characteristics of the included diabetic patients

Variables	Total (*n* = 1914)	No periodontitis group (*n* = 633)	Periodontitis group (*n* = 1281)	Statistics	*P*
Retinol intake, *n* (%)				χ^2^ = 0.53	0.465
<336.46 mcg	1035 (49.96)	339 (48.39)	696 (50.97)		
≥336.46 mcg	879 (50.04)	294 (51.61)	585 (49.03)		
α-carotene intake, *n* (%)				χ^2^ = 4.39	0.036
<55.82 mcg	985 (49.70)	298 (45.63)	687 (52.33)		
≥55.82 mcg	929 (50.30)	335 (54.37)	594 (47.67)		
β-carotene intake, *n* (%)				χ^2^ = 0.01	0.919
<841.49 mcg	978 (49.86)	315 (50.07)	663 (49.72)		
≥841.49 mcg	936 (50.14)	318 (49.93)	618 (50.28)		
β-cryptoxanthin intake, *n* (%)				χ^2^ = 0.02	0.880
<30.13 mcg	989 (49.92)	321 (50.17)	668 (49.76)		
≥30.13 mcg	925 (50.08)	312 (49.83)	613 (50.24)		
Lutein + zeaxanthin intake, *n* (%)				χ^2^ = 4.42	0.036
<795.95 mcg	992 (49.98)	304 (46.09)	688 (52.49)		
≥795.95 mcg	922 (50.02)	329 (53.91)	593 (47.51)		
Lycopene intake, *n* (%)				χ^2^ = 0.09	0.768
<1617.26 mcg	985 (49.96)	315 (49.48)	670 (50.27)		
≥1617.26 mcg	929 (50.04)	318 (50.52)	611 (49.73)		
Age, years, Mean (SE)	58.34 (0.38)	55.04 (0.56)	60.48 (0.46)	*t* = −7.63	<0.001
Gender, *n* (%)				χ^2^ = 21.98	<0.001
Male	996 (52.68)	253 (43.44)	743 (58.64)		
Female	918 (47.32)	380 (56.56)	538 (41.36)		
Race, *n* (%)				χ^2^ = 20.34	<0.001
Non-Hispanic White	654 (57.89)	264 (64.95)	390 (53.33)		
Non-Hispanic Black	506 (15.46)	158 (13.58)	348 (16.68)		
Other	754 (26.65)	211 (21.47)	543 (29.99)		
Education, *n* (%)				χ^2^ = 22.77	<0.001
Less than high school	593 (22.65)	124 (14.97)	469 (27.62)		
High school graduate/GED or equivalent	426 (23.69)	127 (23.66)	299 (23.71)		
Above high school	895 (53.66)	382 (61.37)	513 (48.67)		
Poverty-to-income ratio, *n* (%)				χ^2^ = 14.83	<0.001
≤1.0	382 (15.21)	91 (10.46)	291 (18.28)		
>1.0	1373 (78.47)	493 (84.05)	880 (74.86)		
Unknown	159 (6.33)	49 (5.50)	110 (6.87)		
Smoking, *n* (%)				χ^2^ = 21.02	<0.001
No	1028 (51.91)	394 (59.99)	634 (46.69)		
Yes	886 (48.09)	239 (40.01)	647 (53.31)		
Drinking, *n* (%)				χ^2^ = 1.06	0.590
No	662 (28.48)	216 (26.88)	446 (29.52)		
<1 time/week	839 (46.49)	281 (46.88)	558 (46.24)		
≥1 time/week	413 (25.03)	136 (26.24)	277 (24.25)		
Physical activity, *n* (%)				χ^2^ = 10.49	0.005
<450 MET·min/week	249 (12.97)	85 (15.56)	164 (11.30)		
≥450 MET·min/week	986 (51.82)	346 (54.59)	640 (50.02)		
Unknown	679 (35.21)	202 (29.86)	477 (38.68)		
Dental implants, *n* (%)				χ^2^ = 9.99	0.002
No	1871 (97.67)	609 (96.08)	1262 (98.70)		
Yes	43 (2.33)	24 (3.92)	19 (1.30)		
Diabetic retinopathy, *n* (%)				χ^2^ = 0.61	0.437
No	1705 (90.88)	574 (91.72)	1131 (90.34)		
Yes	209 (9.12)	59 (8.28)	150 (9.66)		
Chronic kidney disease, *n* (%)				χ^2^ = 1.13	0.287
No	1739 (92.06)	585 (93.08)	1154 (91.39)		
Yes	175 (7.94)	48 (6.92)	127 (8.61)		
Hypertension, *n* (%)				χ^2^ = 17.66	<0.001
No	465 (24.88)	183 (31.57)	282 (20.56)		
Yes	1449 (75.12)	450 (68.43)	999 (79.44)		
Dyslipidaemia, *n* (%)				χ^2^ = 0.04	0.851
No	185 (7.91)	74 (7.74)	111 (8.02)		
Yes	1729 (92.09)	559 (92.26)	1170 (91.98)		
Cardiovascular disease, *n* (%)				χ^2^ = 3.09	0.079
No	1164 (61.72)	403 (64.82)	761 (59.72)		
Yes	750 (38.28)	230 (35.18)	520 (40.28)		
Hepatitis, *n* (%)				χ^2^ = 22.00	<0.001
No	1706 (92.28)	588 (95.97)	1118 (89.89)		
Yes	208 (7.72)	45 (4.03)	163 (10.11)		
Autoimmune disease, *n* (%)				χ^2^ = 0.09	0.769
No	1704 (90.04)	561 (89.74)	1143 (90.24)		
Yes	210 (9.96)	72 (10.26)	138 (9.76)		
BMI, kg/m^2^, Mean (SE)	33.05 (0.22)	33.64 (0.40)	32.66 (0.28)	*t* = 1.94	0.058
Waist circumference, cm, Mean (SE)	110.88 (0.54)	111.03 (0.84)	110.78 (0.72)	*t* = 0.23	0.818
White blood cell count, 1000 cells/μL, Mean (SE)	7.63 (0.08)	7.57 (0.16)	7.67 (0.08)	*t* = −0.58	0.564
Total energy, kcal, Mean (SE)	2014.56 (25.09)	2009.87 (45.26)	2017.60 (36.38)	*t* = −0.12	0.904
Total fat, gm, Mean (SE)	80.52 (1.20)	80.73 (2.09)	80.39 (1.82)	*t* = 0.11	0.913
Number of missing teeth, *n* (%)				χ^2^ = 119.30	<0.001
≤5	1009 (60.17)	433 (75.35)	576 (50.36)		
>5	905 (39.83)	200 (24.65)	705 (49.64)		
Frequency of using dental floss, *n* (%)				χ^2^ = 8.85	0.003
<3 times/week	994 (49.36)	271 (42.27)	723 (53.94)		
≥3 times/week	920 (50.64)	362 (57.73)	558 (46.06)		
Antibiotics, *n* (%)				χ^2^ = 1.29	0.256
No	1855 (95.49)	607 (94.13)	1248 (96.37)		
Yes	59 (4.51)	26 (5.87)	33 (3.63)		
Anti-diabetic drugs, *n* (%)				χ^2^ = 0.24	0.625
No	712 (37.38)	246 (38.36)	466 (36.75)		
Yes	1202 (62.62)	387 (61.64)	815 (63.25)		

SE: standard error; GED: general educational development; MET: metabolic equivalent.

### Association between dietary carotenoid intake and periodontitis

After adjusting for age, gender, race, education, smoking, dental implants, hepatitis, and the number of missing teeth (Supplementary Table S2), α-carotene intake ≥55.82 mcg was associated with lower odds of periodontitis than α-carotene intake <55.82 mcg (OR = 0.70, 95%CI: 0.53–0.91, *P* = 0.010); lutein and zeaxanthin intake ≥795.95 mcg was associated with decreased odds of periodontitis than lutein and zeaxanthin intake <795.95 mcg (OR = 0.75, 95%CI: 0.57–0.98, *P* = 0.039); no significant association was observed between dietary intake of β-carotene, β-cryptoxanthin and lycopene and the odds of periodontitis in diabetic patients (all *P*>0.05) (Table 2). As illustrated in Supplementary Table S3, compared with α-carotene intake <55.82 mcg, α-carotene intake ≥55.82 mcg was associated with reduced odds of mild or moderate periodontitis (OR = 0.73, 95%CI: 0.55–0.98, *P* = 0.037). There were no significant association between the intake of α-carotene, β-carotene, β-cryptoxanthin, lutein and zeaxanthin, and lycopene and the odds of severe periodontitis (all *P*>0.05).

**Table 2.** Association between dietary carotenoid intake and periodontitis

Variables	Model I		Model II		Model III	
	OR (95%CI)	*P*	OR (95%CI)	*P*	OR (95%CI)	*P*
Retinol intake						
<336.46 mcg	Ref		Ref		Ref	
≥336.46 mcg	0.90 (0.68–1.20)	0.470	0.95 (0.70–1.28)	0.714	0.97 (0.72–1.31)	0.840
α-carotene intake						
<55.82 mcg	Ref		Ref		Ref	
≥55.82 mcg	0.76 (0.59–0.99)	0.039	0.66 (0.51–0.86)	0.003	0.70 (0.53–0.91)	0.010
β-carotene intake						
<841.49 mcg	Ref		Ref		Ref	
≥841.49 mcg	1.01 (0.77–1.34)	0.920	0.90 (0.66–1.22)	0.491	0.95 (0.70–1.29)	0.761
β-cryptoxanthin intake						
<30.13 mcg	Ref		Ref		Ref	
≥30.13 mcg	1.02 (0.82–1.27)	0.881	0.88 (0.71–1.09)	0.245	0.92 (0.73–1.16)	0.464
Lutein + zeaxanthin intake						
<795.95 mcg	Ref		Ref		Ref	
≥795.95 mcg	0.77 (0.61–0.99)	0.038	0.68 (0.53–0.88)	0.004	0.75 (0.57–0.98)	0.039
Lycopene intake						
<1617.26 mcg	Ref		Ref		Ref	
≥1617.26 mcg	0.97 (0.78–1.20)	0.770	0.94 (0.76–1.18)	0.608	0.97 (0.78–1.21)	0.792

Model I, a univariate model;

Model II, a multivariate model, adjusted for age, gender, and race;

Model III, a multivariate model, adjusted for age, gender, race, education, smoking, dental implants, hepatitis, and the number of missing teeth.

Ref: reference.

### Association between dietary carotenoid intake and periodontitis in different subpopulations

#### Age

In diabetic patients aged ≥60 years, α-carotene intake ≥55.82 mcg was associated with reduced odds of periodontitis than α-carotene intake <55.82 mcg after controlling for gender, race, education, smoking, dental implants, hepatitis, and the number of missing teeth (OR = 0.55, 95%CI: 0.38–0.79, *P* < 0.01) (Table 3).

**Table 3.** Association between dietary carotenoid intake and periodontitis by age and gender

	OR (95%*CI*)					
Variables	Subgroup I: Age				Subgroup II: Gender	
Subgroup	Age<60 (*n* = 889)		Age≥60 (*n* = 1025)		Male (*n* = 996)	Female (*n* = 918)
Retinol intake, *n* (%)						
<336.46 mcg	Ref		Ref		Ref	Ref
≥336.46 mcg	0.95 (0.64–1.43)		1.06 (0.65–1.74)		1.07 (0.71–1.63)	0.84 (0.57–1.22)
α-carotene intake, *n* (%)						
<55.82 mcg	Ref		Ref		Ref	Ref
≥55.82 mcg	0.94 (0.62–1.42)		0.55 (0.38–0.79)**		0.69 (0.47–1.02)	0.69 (0.46–1.04)
β-carotene intake, *n* (%)						
<841.49 mcg	Ref		Ref		Ref	Ref
≥841.49 mcg	1.15 (0.76–1.74)		0.86 (0.63–1.16)		1.04 (0.66–1.65)	0.86 (0.58–1.27)
β-cryptoxanthin intake, *n* (%)						
<30.13 mcg	Ref		Ref		Ref	Ref
≥30.13 mcg	0.77 (0.52–1.12)		1.15 (0.80–1.64)		0.95 (0.62–1.44)	0.82 (0.56–1.20)
Lutein + zeaxanthin intake, *n* (%)						
<795.95 mcg	Ref		Ref		Ref	Ref
≥795.95 mcg	0.72 (0.49–1.06)		0.85 (0.59–1.22)		0.88 (0.61–1.26)	0.62 (0.38–0.99)*
Lycopene intake, *n* (%)						
<1617.26 mcg	Ref		Ref		Ref	Ref
≥1617.26 mcg	1.02 (0.70–1.49)		0.99 (0.71–1.37)		1.02 (0.66–1.57)	0.89 (0.60–1.31)
Subgroup	Subgroup III: Number of missing teeth				Subgroup IV: Cardiovascular disease	
	≤5 (*n* = 1009)	6–10 (*n* = 372)	11–15 (*n* = 200)	>15 (*n* = 333)	No (*n* = 1164)	Yes (*n* = 750)
Retinol intake, *n* (%)						
<336.46 mcg	Ref	Ref	Ref	Ref	Ref	Ref
≥336.46 mcg	1.02 (0.71–1.46)	0.90 (0.36–2.22)	0.78 (0.23–2.66)	0.99 (0.47–2.08)	0.95 (0.66–1.37)	1.01 (0.64–1.58)
α-carotene intake, *n* (%)						
<55.82 mcg	Ref	Ref	Ref	Ref	Ref	Ref
≥55.82 mcg	0.64 (0.46–0.89)**	0.78 (0.41–1.49)	0.82 (0.34–1.99)	0.70 (0.34–1.44)	0.61 (0.42–0.88)**	0.80 (0.53–1.23)
β-carotene intake, *n* (%)						
<841.49 mcg	Ref	Ref	Ref	Ref	Ref	Ref
≥841.49 mcg	0.79 (0.51–1.22)	1.13 (0.50–2.56)	0.86 (0.35–2.11)	1.63 (0.79–3.35)	0.92 (0.62–1.37)	0.95 (0.59–1.52)
β-cryptoxanthin intake, *n* (%)						
<30.13 mcg	Ref	Ref	Ref	Ref	Ref	Ref
≥30.13 mcg	0.86 (0.59–1.26)	0.81 (0.40–1.64)	0.68 (0.19–2.48)	1.22 (0.68–2.19)	0.86 (0.63–1.17)	0.98 (0.61–1.56)
Lutein + zeaxanthin intake, *n* (%)						
<795.95 mcg	Ref	Ref	Ref	Ref	Ref	Ref
≥795.95 mcg	0.68 (0.47–0.97)*	0.77 (0.39–1.55)	0.55 (0.19–1.65)	1.50 (0.69–3.26)	0.61 (0.42–0.88)**	0.98 (0.63–1.50)
Lycopene intake, *n* (%)						
<1617.26 mcg	Ref	Ref	Ref	Ref	Ref	Ref
≥1617.26 mcg	0.94 (0.70–1.26)	1.19 (0.67–2.11)	0.74 (0.25–2.16)	1.05 (0.51–2.17)	0.95 (0.71–1.29)	0.98 (0.66–1.45)
Subgroup	Subgroup V: Smoking				Subgroup VI: Anti-diabetic drugs	
	No (*n* = 1028)		Yes (*n* = 886)		No (*n* = 712)	Yes (*n* = 1202)
Retinol intake, *n* (%)						
<336.46 mcg	Ref		Ref		Ref	Ref
≥336.46 mcg	1.15 (0.76–1.73)		0.71 (0.47–1.08)		0.95 (0.55–1.65)	0.96 (0.69–1.34)
α-carotene intake, *n* (%)						
<55.82 mcg	Ref		Ref		Ref	Ref
≥55.82 mcg	0.69 (0.49–0.96)*		0.69 (0.45–1.06)		0.71 (0.41–1.24)	0.70 (0.51–0.95)*
β-carotene intake, *n* (%)						
<841.49 mcg	Ref		Ref		Ref	Ref
≥841.49 mcg	0.81 (0.52–1.25)		1.12 (0.74–1.70)		0.85 (0.50–1.43)	1.02 (0.74–1.41)
β-cryptoxanthin intake, *n* (%)						
<30.13 mcg	Ref		Ref		Ref	Ref
≥30.13 mcg	0.71 (0.54–0.93)*		1.15 (0.69–1.93)		0.90 (0.58–1.39)	0.96 (0.74–1.23)
Lutein + zeaxanthin intake, *n* (%)						
<795.95 mcg	Ref		Ref		Ref	Ref
≥795.95 mcg	0.58 (0.40–0.83)**		0.92 (0.57–1.46)		0.63 (0.39–1.03)	0.81 (0.59–1.13)
Lycopene intake, *n* (%)						
<1617.26 mcg	Ref		Ref		Ref	Ref
≥1617.26 mcg	1.00 (0.73–1.37)		0.99 (0.70–1.40)		0.92 (0.59–1.42)	0.96 (0.69–1.33)

In age subgroups, gender, race, education, smoking, dental implants, hepatitis, and the number of missing teeth were adjusted for;

In gender subgroups, age, race, education, smoking, dental implants, hepatitis, and the number of missing teeth were adjusted for.

In number of missing teeth subgroups, age, gender, race, education, smoking, dental implants, and hepatitis were adjusted for.

In cardiovascular disease subgroups, age, gender, race, education, smoking, dental implants, hepatitis, and the number of missing teeth were adjusted for.

In smoking subgroups, age, gender, race, education, dental implants, hepatitis, and the number of missing teeth were adjusted for.

In anti-diabetic drug subgroups, age, gender, race, education, smoking, dental implants, hepatitis, and the number of missing teeth were adjusted for.

Ref: reference.

* : *P* < 0.05,

** : *P* < 0.01.

#### Gender

Diabetic females with lutein and zeaxanthin intake ≥795.95 mcg had lower odds of periodontitis than those with lutein and zeaxanthin intake <795.95 mcg following adjustment for age, race, education, smoking, dental implants, hepatitis, and the number of missing teeth (OR = 0.62, 95%CI: 0.38–0.99, *P* < 0.05) (Table 3).

#### Number of missing teeth

For patients with ≤ 5 missing teeth, α-carotene intake ≥55.82 mcg and lutein and zeaxanthin intake ≥795.95 mcg were associated with decreased odds of periodontitis in contrast to α-carotene intake <55.82 mcg (OR = 0.64, 95%CI: 0.46–0.89, *P* < 0.01) and lutein and zeaxanthin intake <795.95 mcg (OR = 0.68, 95%CI: 0.47–0.97, *P* < 0.05), respectively; no significant association was found between α-carotene, β-carotene, β-cryptoxanthin, lutein and zeaxanthin, and lycopene and the odds of periodontitis in patients with 6–10, 11–15, and >15 missing teeth (all *P*>0.05), after adjusting for age, gender, race, education, smoking, dental implants, and hepatitis (Table 3).

#### Cardiovascular disease

Patients without cardiovascular disease who consumed ≥55.82 mcg of α-carotene and ≥795.95 mcg of lutein and zeaxanthin had lower odds of periodontitis than those who consumed <55.82 mcg of α-carotene (OR = 0.61, 95%CI: 0.42–0.88, *P* < 0.01) and <795.95 mcg of lutein and zeaxanthin (OR = 0.61, 95%CI: 0.42–0.88, *P* < 0.01), separately, after adjusting age, gender, race, education, smoking, dental implants, hepatitis, and the number of missing teeth (Table 3).

#### Smoking

Among non-smokers, dietary intake of α-carotene (OR = 0.69, 95%CI: 0.49–0.96, *P* < 0.01), β-cryptoxanthin (OR = 0.71, 95%CI: 0.54–0.93, *P* < 0.01), and lutein and zeaxanthin (OR = 0.58, 95%CI: 0.40–0.83, *P* < 0.01) was negatively associated with the odds of periodontitis after adjusting age, gender, race, education, dental implants, hepatitis, and the number of missing teeth (Table 3).

#### Anti-diabetic drugs

In patients receiving anti-diabetic drugs, α-carotene intake ≥55.82 mcg was associated with lower odds of periodontitis, as compared with α-carotene intake <55.82 mcg, after adjusting age, gender, race, education, smoking, dental implants, hepatitis, and the number of missing teeth (OR = 0.70, 95%CI: 0.51–0.95, *P* < 0.05) (Table 3).

## Discussion

To the best of our knowledge, this study was the first to investigate the association between dietary intake of five carotenoids and the odds of periodontitis in patients with diabetes. The results demonstrated that higher intake of α-carotene and lutein and zeaxanthin was associated with lower odds of periodontitis in diabetic patients; further, the negative association between carotenoid intake and periodontitis remained significant in patients aged ≥60 years, female patients, patients with ≤ 5 missing teeth, patients without cardiovascular disease, non-smoking patients, and patients receiving anti-diabetic drugs, which may serves as a reference for periodontitis prevention and control in diabetic patients and further in subpopulations.

A study on older Japanese evaluated the relationship between intake of dietary antioxidants, including α-carotene and β-carotene, and periodontal disease, and found that β-carotene at the third tertile was associated with fewer teeth with periodontal disease progression^(7)^. Dodington et al.^(26)^ reported the association between higher dietary intake of β-carotene and greater healing after periodontal procedures in non-smokers. According to a previous review, β-carotene was correlated with the risk of periodontal disease in community-based older people^(27)^. As shown by Zhou et al.^(28)^, dietary retinol intake was negatively associated with periodontitis among US adults. However, no study has probed into the relationship between dietary intake of carotenoids and periodontitis in the diabetic population. The present study thus filled this research gap, and showed that increased intake of α-carotene and lutein and zeaxanthin was associated with decreased odds of periodontitis in people with diabetes. Many diseases, including periodontitis, are characterized by oxidative stress. A recent study found that periodontitis-related human gingival fibroblasts generate more reactive oxygen species (ROS)^(29)^. In periodontitis, ROS has been described as a double-edged sword. Neutrophils produce ROS to eradicate invasive pathogenic microbes in healthy periodontal tissue, but too much ROS can cause cytotoxicity to host cells, and facilitate formation and progression of periodontitis^(30,31)^. The association of α-carotene and lutein and zeaxanthin with periodontitis in diabetes may be brought about by the antioxidant action of these carotenoids, or by their function in immunological regulation^(32,33)^. Walston et al.^(34)^ suggested that interleukin-6 (IL-6), a marker of systemic inflammation, was more likely to be elevated in elderly people who had low levels of a-carotene, which was linked to poor health outcomes. As regards no significant association between β-carotene and periodontitis while the association existed between α-carotene and periodontitis, possible explanations include: (1) although α-carotene is chemically similar to β-carotene, α-carotene has greater potential antioxidant effects^(35)^; (2) α-carotene exhibits a greater apparent bioavailability than β-carotene^(36)^. This study also compared patients with severe periodontitis with those who are completely healthy, and illustrated no significant association between the intake of carotenoids and the odds of severe periodontitis. This may be attributed to a small sample size for analysis (*n* = 341), and for severe cases, the association between carotenoids and periodontitis may be too small to be significant, which required more research to verify.

Furthermore, this study illustrated that in diabetic patients aged ≥60 years, α-carotene intake was inversely associated with periodontitis, and among female patients with diabetes, lutein and zeaxanthin intake was negatively correlated with periodontitis. The majority of older people experience inflammageing, which is featured by raised blood inflammatory marker levels and is highly susceptible to chronic illnesses such as diabetes and periodontitis^(37,38)^. Besides, women are more likely to develop chronic inflammatory disorders than men, and oestrogen may influence T regulatory cell immune response in females^(39)^. These may account for the significant association between carotenoid intake and periodontitis in ≥60 years and female groups. For diabetic patients with ≤ 5 missing teeth, patients without cardiovascular disease, non-smoking patients, and patients receiving anti-diabetic drugs, α-carotene, lutein and zeaxanthin, or β-cryptoxanthin was also found to be related to periodontitis. Over 5 missing teeth, cardiovascular disease, smoking, and uncontrolled diabetes are greatly correlated with the higher incidence and progression of periodontitis^(40–43)^, which might overshadow or mask the relationship between carotenoid intake and periodontitis. Importantly, these groups of diabetic patients should pay more attention to their oral health, and higher intake of α-carotene, lutein and zeaxanthin, or β-cryptoxanthin may become a reference strategy in hindering the occurrence and development of periodontitis, which requires future studies to confirm.

In this study, a nationally representative sample was used to first explore the relationship between dietary carotenoids and periodontitis in people with diabetes, and periodontal examinations were relatively comprehensive (covering 28 teeth, with 6 sites per tooth). Participants with no or mild periodontitis were included into the no periodontitis group, in order to mitigate the risk of bias due to a potentially excessive prevalence of mild periodontitis in the population^(20)^, and individuals with mild periodontitis is not as significant as moderate or severe according to the CDC-APA classification. Based on our findings, in diabetes, patients could raise their awareness of healthy eating, take food rich in α-carotene (such as carrots, squash, and broccoli) and lutein and zeaxanthin (such as kale, honey melon, kiwi fruit, and egg yolk) or corresponding supplements, and undergo regular oral examinations to reduce the odds of periodontitis occurrence and progression; patients with periodontitis could pay attention to their diet and increase their intake of α-carotene and lutein and zeaxanthin. Some limitations should be acknowledged in result interpretation. First, this study had a cross-sectional design, and the causal relationship of carotenoid intake and periodontitis could not be determined, which needs further exploration through cohort studies. Second, dietary intake of carotenoids was obtained from the 24-hour dietary recall interview, which may have been affected by recall bias. Besides, one millimetre that the dental clinician could easily have mismeasured was used to delimit whether a patient is in the periodontitis or healthy group. Of note, periodontal examinations in the NHANES were conducted by trained dental examiners and the quality of data was assured and controlled (https://wwwn.cdc.gov/Nchs/Nhanes/2009-2010/OHXPER_F.htm). Third, the NHANES database did not collect information on dental plaque, gum treatment, data on the length of time the person has been living with diabetes, the type of diabetes, and diabetes control, so the impact of these factors was not considered in our analysis. Due to a lack of relevant information in the database, we could not know what happened to the individuals with 26 teeth missing whose last two teeth were healthy, which indicated that future investigations should improve their reporting of the history of periodontitis. Additionally, because of limited available data from the NHANES, future large-scale studies can be performed to investigate the dose-response association between carotenoid intake and periodontitis in diabetic patients.

In conclusion, among diabetic patients, intake of α-carotene and lutein and zeaxanthin was inversely associated with the odds of periodontitis. The association between dietary carotenoid intake and periodontitis differed by age, gender, the number of missing teeth, cardiovascular disease, smoking, and anti-diabetic drugs. Future studies are warranted to support these findings and to investigate causality.

## Supporting information

Li et al. supplementary material Li et al. supplementary material

## Supplementary material

To view supplementary material for this article, please visit https://doi.org/10.1017/jns.2023.116
