# Supplementary material for: Association between carotenoid intake and periodontitis in diabetic patients
Source: J Nutr Sci. 2024 Feb 28;13:e11. doi: 10.1017/jns.2023.116 (PMC10988174; doi:10.1017/jns.2023.116)
Supplement: Li et al. supplementary material [file S2048679023001167sup001.docx]

Supplementary Table S1 Sensitivity analysis for differences between the data before and after imputation.

| Variables | Before imputation | After imputation | Statistics | *P* |
| --- | --- | --- | --- | --- |
| Education, n (%) |  |  | χ^2^=2.69 | 0.261 |
| Less than high school | 591 (22.62) | 593 (22.65) |  |  |
| High school graduate/GED or equivalent | 424 (23.69) | 426 (23.69) |  |  |
| Above high school | 895 (53.69) | 895 (53.66) |  |  |
| Frequency of using dental floss, n (%) |  |  | χ^2^=2.19 | 0.139 |
| < 3 times/week | 973 (49.14) | 994 (49.36) |  |  |
| ≥ 3 times/week | 918 (50.86) | 920 (50.64) |  |  |
| Body Mass Index, kg/m^2^, Mean (SE) | 33.05 (0.22) | 33.05 (0.22) | t=-0.22 | 0.825 |
| Waist circumference, cm, Mean (SE) | 110.65 (0.53) | 110.88 (0.54) | t=-1.92 | 0.060 |
| White blood cell count, 1000 cells/μL, Mean (SE) | 7.63 (0.08) | 7.63 (0.08) | t=0.25 | 0.806 |
| Smoking, n (%) |  |  | χ^2^=1.00 | 0.317 |
| No | 1028 (51.94) | 1028 (51.91) |  |  |
| Yes | 885 (48.06) | 886 (48.09) |  |  |
| Drinking, n (%) |  |  | χ^2^=2.30 | 0.316 |
| No | 630 (28.35) | 662 (28.48) |  |  |
| < 1 time/week | 808 (46.35) | 839 (46.49) |  |  |
| ≥ 1 time/week | 400 (25.30) | 413 (25.03) |  |  |
| Chronic kidney disease, n (%) |  |  | χ^2^=0.47 | 0.493 |
| No | 1658 (92.16) | 1739 (92.06) |  |  |
| Yes | 161 (7.84) | 175 (7.94) |  |  |
| Hepatitis, n (%) |  |  | χ^2^=2.26 | 0.132 |
| No | 1615 (92.69) | 1706 (92.28) |  |  |
| Yes | 190 (7.31) | 208 (7.72) |  |  |

SE: standard error; GED: general educational development.

Supplementary Table S2 Weighted univariate logistic regression analysis for confounders.

| Variables | OR (95%CI) | *P* |
| --- | --- | --- |
| Age | 1.04 (1.03-1.05) | <0.001 |
| Gender |  |  |
| Male | Ref |  |
| Female | 0.54 (0.41-0.71) | <0.001 |
| Race |  |  |
| Non-Hispanic White | Ref |  |
| Non-Hispanic Black | 1.50 (1.11-2.02) | 0.010 |
| Other | 1.70 (1.29-2.24) | <0.001 |
| Education |  |  |
| Less than high school | Ref |  |
| High school graduate/GED or equivalent | 0.54 (0.36-0.81) | 0.004 |
| Above high school | 0.43 (0.30-0.61) | <0.001 |
| Poverty-to-income ratio |  |  |
| ≤ 1.0 | Ref |  |
| > 1.0 | 0.51 (0.35-0.74) | <0.001 |
| Unknown | 0.72 (0.37-1.37) | 0.305 |
| Smoking |  |  |
| No | Ref |  |
| Yes | 1.71 (1.34-2.18) | <0.001 |
| Drinking |  |  |
| No | Ref |  |
| < 1 time/week | 0.90 (0.68-1.18) | 0.430 |
| ≥ 1 time/week | 0.84 (0.59-1.20) | 0.333 |
| Physical activity |  |  |
| < 450 MET·min/week | Ref |  |
| ≥ 450 MET·min/week | 1.26 (0.95-1.68) | 0.111 |
| Unknown | 1.78 (1.17-2.71) | 0.008 |
| Dental implants |  |  |
| No | Ref |  |
| Yes | 0.32 (0.15-0.70) | 0.005 |
| Diabetic retinopathy |  |  |
| No | Ref |  |
| Yes | 1.19 (0.76-1.84) | 0.439 |
| Chronic kidney disease |  |  |
| No | Ref |  |
| Yes | 1.27 (0.81-1.98) | 0.293 |
| Hypertension |  |  |
| No | Ref |  |
| Yes | 1.78 (1.35-2.35) | <0.001 |
| Dyslipidemia |  |  |
| No | Ref |  |
| Yes | 0.96 (0.64-1.45) | 0.852 |
| Cardiovascular disease |  |  |
| No | Ref |  |
| Yes | 1.24 (0.97-1.59) | 0.083 |
| Hepatitis |  |  |
| No | Ref |  |
| Yes | 2.68 (1.71-4.20) | <0.001 |
| Autoimmune disease |  |  |
| No | Ref |  |
| Yes | 0.95 (0.65-1.38) | 0.769 |
| Body mass index | 0.98 (0.96-1.00) | 0.051 |
| Waist circumference | 1.00 (0.99-1.01) | 0.817 |
| White blood cell count | 1.02 (0.95-1.10) | 0.571 |
| Total energy | 1.00 (1.00-1.00) | 0.905 |
| Total fat | 1.00 (1.00-1.00) | 0.913 |
| Number of missing teeth |  |  |
| ≤ 5 | Ref |  |
| > 5 | 3.01 (2.38-3.81) | <0.001 |
| Frequency of using dental floss |  |  |
| < 3 times/week | Ref |  |
| ≥ 3 times/week | 0.63 (0.46-0.86) | 0.004 |
| Antibiotics |  |  |
| No | Ref |  |
| Yes | 0.60 (0.25-1.48) | 0.263 |
| Anti-diabetic drug |  |  |
| No | Ref |  |
| Yes | 1.07 (0.81-1.42) | 0.627 |

OR: odds ratio; CI: confidence interval; Ref: reference; GED: general educational development; MET: metabolic equivalent.

Supplementary Table S3 Association between dietary carotenoid intake and periodontitis (mild or moderate, severe).

| Variables | No periodontitis | Mild or Moderate periodontitis | | Severe periodontitis | |
| --- | --- | --- | --- | --- | --- |
|  |  | OR (95%CI) | *P* | OR (95%CI) | *P* |
| Retinol intake |  |  |  |  |  |
| < 336.46 mcg |  |  |  |  |  |
| ≥ 336.46 mcg | Ref | 1.01 (0.71-1.43) | 0.976 | 0.92 (0.56-1.52) | 0.739 |
| α-carotene intake |  |  |  |  |  |
| < 55.82 mcg |  |  |  |  |  |
| ≥ 55.82 mcg | Ref | 0.73 (0.55-0.98) | 0.037 | 0.63 (0.38-1.04) | 0.070 |
| β-carotene intake |  |  |  |  |  |
| < 841.49 mcg |  |  |  |  |  |
| ≥ 841.49 mcg | Ref | 1.03 (0.76-1.41) | 0.825 | 0.98 (0.63-1.53) | 0.942 |
| β-cryptoxanthin intake |  |  |  |  |  |
| < 30.13 mcg |  |  |  |  |  |
| ≥ 30.13 mcg | Ref | 1.03 (0.80-1.33) | 0.824 | 1.02 (0.68-1.52) | 0.936 |
| Lutein + zeaxanthin intake |  |  |  |  |  |
| < 795.95 mcg |  |  |  |  |  |
| ≥ 795.95 mcg | Ref | 0.87 (0.66-1.15) | 0.327 | 0.88 (0.60-1.28) | 0.489 |
| Lycopene intake |  |  |  |  |  |
| < 1617.26 mcg |  |  |  |  |  |
| ≥ 1617.26 mcg | Ref | 1.05 (0.83-1.31) | 0.688 | 1.03 (0.62-1.69) | 0.910 |

Age, gender, race, education, smoking, dental implants, hepatitis, and the number of missing teeth were adjusted for.

OR: odds ratio; CI: confidence interval; Ref: reference.
